# Effects of Arsenic

**Published:** 1890-07-12

**Authors:** 


### THERAPEUTICS.

### EFFECTS OF ARSENIC.

Some time ago the reports of two cases of pigmentation of the skin after the use of arsenic were published by Dr. Haffner, of Berlin. In a quite recent number of the *New York Medical Record*, Dr. Wehlau details an instance coming under his observation. A female was suffering from *anæmia progressiva perniciosa*, for which five drops of Fowler's solution three times a day was prescribed. After having taken the arsenic three weeks she developed a discolouration of the skin, that reminded one of a "far advanced case of Addison's disease," the pigmentation being most pronounced on the abdomen, thighs, face, and arms. The greatest intensity of pigmentation lasted about ten days, and then a fine whitish desquamation occurred, after which the skin assumed its natural colour. Dr. William Leszynsky has also reported a similar case at the Academy of Medicine, New York, in April of last year.

Now the ordinary symptoms produced by a long course of arsenic are usually evidenced first in the eyes and stomach. Itching, smarting, and suffusion of the eyes occur, and the lids, especially the lower, may become slightly œdematous; and there may be a sensation of soreness at the pit of the stomach or colicky pains in the bowels. It has also been frequently noticed that the skin becomes dry and dirty looking, and a slight branniness has presented more marked where the person is most covered with clothes. We do not recollect, however, ever having seen anything resembling what might be called pigmentation of the skin, as described by Dr. Wehlau. And as pigmentation of the skin as a result of arsenic is not mentioned by Ringer, and various other authors, who have written on the effects of medicines, it may be regarded as certain that this drug does not very frequently cause pigmentation. It must be recollected that pigmentation of the skin occurs in connection with various conditions. *Melaena palpebrarum* accompanies every menstrual period in some females. In pregnancy *melaena* of the areola of the nipple is an ordinary occurrence. Melaena is a constant attendant on prurigo. And it is well known to be frequently associated with Addison's disease. A leaden or slate-coloured hue resulting from a prolonged use of the salts of silver has often been noticed, and this action of silver salts has been utilised for the treatment of the disease known in the East as *leucoderma*, or white skin; an affection which has often been confounded with leprosy, but which is perfectly distinct from leprosy. As regards pigmentation following the use of arsenic medicinally, we are somewhat sceptical. Pigmentation may occur after the use of arsenic, but it does not follow that it is caused by the arsenic. The drug when used judiciously is frequently so beneficial in a variety of morbid conditions that anything militating against its employment should be received with caution. It is, however, well to recollect that cases of pigmentation after taking arsenic have now been reported; also that such pigmentation is not, like the discolouration produced by silver salts, of a permanent character.

